# Within-patient correspondence of amyloid-β and intrinsic network connectivity in Alzheimer’s disease

**DOI:** 10.1093/brain/awu103

**Published:** 2014-04-26

**Authors:** Nicholas Myers, Lorenzo Pasquini, Jens Göttler, Timo Grimmer, Kathrin Koch, Marion Ortner, Julia Neitzel, Mark Mühlau, Stefan Förster, Alexander Kurz, Hans Förstl, Claus Zimmer, Afra M. Wohlschläger, Valentin Riedl, Alexander Drzezga, Christian Sorg

**Affiliations:** 1 Department of Neuroradiology, Technische Universität München, Ismaningerstr. 22, 81675 Munich, Germany; 2 TUM-Neuroimaging Centre, Technische Universität München, Ismaningerstr. 22, 81675 Munich, Germany; 3 Department of Experimental Psychology, Oxford University, 9 South Parks Road, Oxford OX1 3UD, UK; 4 Department of Psychiatry, Technische Universität München, Ismaningerstr. 22, 81675 Munich, Germany; 5 Department of Neurology of Klinikum rechts der Isar, Technische Universität München, Ismaningerstr. 22, 81675 Munich, Germany; 6 Department of Nuclear Medicine, Technische Universität München, Ismaningerstr. 22, 81675 Munich, Germany; 7 Department of Nuclear Medicine, University of Cologne, Kerpener Straße 62, 50937 Köln, Germany

**Keywords:** Alzheimer’s disease, amyloid-β plaques, intrinsic connectivity, resting-state functional MRI, PiB-PET

## Abstract

There is striking overlap between the spatial distribution of amyloid-β pathology in patients with Alzheimer’s disease and the spatial distribution of high intrinsic functional connectivity in healthy persons. This overlap suggests a mechanistic link between amyloid-β and intrinsic connectivity, and indeed there is evidence in patients for the detrimental effects of amyloid-β plaque accumulation on intrinsic connectivity in areas of high connectivity in heteromodal hubs, and particularly in the default mode network. However, the observed spatial extent of amyloid-β exceeds these tightly circumscribed areas, suggesting that previous studies may have underestimated the negative impact of amyloid-β on intrinsic connectivity. We hypothesized that the known positive baseline correlation between patterns of amyloid-β and intrinsic connectivity may mask the larger extent of the negative effects of amyloid-β on connectivity. Crucially, a test of this hypothesis requires the within-patient comparison of intrinsic connectivity and amyloid-β distributions. Here we compared spatial patterns of amyloid-β-plaques (measured by Pittsburgh compound B positron emission tomography) and intrinsic functional connectivity (measured by resting-state functional magnetic resonance imaging) in patients with prodromal Alzheimer’s disease via spatial correlations in intrinsic networks covering fronto-parietal heteromodal cortices. At the global network level, we found that amyloid-β and intrinsic connectivity patterns were positively correlated in the default mode and several fronto-parietal attention networks, confirming that amyloid-β aggregates in areas of high intrinsic connectivity on a within-network basis. Further, we saw an internetwork gradient of the magnitude of correlation that depended on network plaque-load. After accounting for this globally positive correlation, local amyloid-β-plaque concentration in regions of high connectivity co-varied negatively with intrinsic connectivity, indicating that amyloid-β pathology adversely reduces connectivity anywhere in an affected network as a function of local amyloid-β-plaque concentration. The local negative association between amyloid-β and intrinsic connectivity was much more pronounced than conventional group comparisons of intrinsic connectivity would suggest. Our findings indicate that the negative impact of amyloid-β on intrinsic connectivity in heteromodal networks is underestimated by conventional analyses. Moreover, our results provide first within-patient evidence for correspondent patterns of amyloid-β and intrinsic connectivity, with the distribution of amyloid-β pathology following functional connectivity gradients within and across intrinsic networks.

## Abstract (precis)

The cortical distribution of amyloid-β plaques in Alzheimer’s disease strikingly resembles frontal-parietal intrinsic functional connectivity networks. Using a novel method to trace the distribution of amyloid-β plaques within single patients, Myers et al. reveal a marked negative effect on intrinsic connectivity in several networks that have not typically been investigated.

## Introduction

Alzheimer’s disease is tightly associated with amyloid-β pathology. Aberrant clearance of amyloid-β precursor protein is thought to be a critical initial event in the disease’s pathogenesis, leading to amyloid-β peptide accumulation and plaque formation 20 to 30 years before cognitive symptoms arise (Selkoe, 2002; Jack *et al.*, 2010; Bateman *et al.*, 2012). Typically, the deposition of plaques has been associated with the default mode network (DMN; Buckner *et al.*, 2005; Sperling *et al.*, 2009), a set of frontal and parietal midline structures with high metabolic activity that are coupled through high intrinsic functional connectivity (i.e. synchronous ongoing activity, where regions that are more strongly synchronized will exhibit higher connectivity). This association is mostly due to the apparent spatial overlap between the DMN and the average deposition of amyloid across the cortex (e.g. Buckner *et al.*, 2005, 2009; Vlassenko *et al.*, 2010; Mormino *et al.*, 2011). The overlap has led to the proposal that in Alzheimer’s disease; (i) amyloid-β-dependent neurodegeneration progresses along network boundaries, spreading to functionally connected areas rather than to spatially contiguous but less connected neighbours (He *et al.*, 2008; Seeley *et al.*, 2009; Bero *et al.*, 2012; Zhou *et al.*, 2012); and that (ii) amyloid-β pathology is accelerated by local stress caused by a lifetime of increased metabolism and intrinsic activity and connectivity (Greicius *et al.*, 2004; Buckner *et al.*, 2005, 2009; Bero *et al.*, 2011; Drzezga *et al.*, 2011). Given the widespread and well-known distribution of amyloid-β outside the DMN (Lehmann *et al.*, 2013*a*, *b*; Sepulcre *et al.*, 2013), and the well-characterized deficits in functional connectivity of other intrinsic networks covering heteromodal areas (Sorg *et al.*, 2007; Brier *et al.*, 2012; Li *et al.*, 2012; see also Sorg *et al.*, 2012), the latter point has been reformulated in a model that supposes effects of amyloid-β in hetermodal (i.e. fronto-parietal) functional networks in general (Jagust and Mormino, 2011). However, this revised model has not been empirically validated.

Critically, the observed spatial overlap implies two concurrent relationships between amyloid-β pathology and intrinsic connectivity. On the one hand, animal studies have linked amyloid-β to reduced intrinsic activity and connectivity (Busche *et al.*, 2008; Bero *et al.*, 2011), a finding that has been repeatedly corroborated in the human DMN (Sheline *et al.*, 2010; Mormino *et al.*, 2011; for a review see Sheline and Raichle, 2013). On the other hand, the spatial overlap also suggests the possibility of a more graded ‘positive’ relationship: wherever intrinsic connectivity is high, amyloid-β pathology tends to be high as well. This has been established for the spatial distribution of amyloid-β and intrinsic connectivity across subjects (Buckner *et al.*, 2009; Drzezga *et al.*, 2011; Bero *et al.*, 2012), but should also apply intra-individually. However, most studies to date have observed these two relationships in isolation. This could have led to an overlooked confound: assume Patient X with particularly high lifetime (or baseline) intrinsic connectivity is prone to stronger amyloid-β plaque accumulation. In turn, the stronger accumulation leads to an increased reduction in connectivity. Compared to Patient Y with lower baseline connectivity (resulting in less plaque accumulation, with a less severe impact on connectivity), the higher baseline connectivity in Patient X could confound measures of that patient’s amyloid-β-induced connectivity ‘reduction’, making them appear less severe than they are. By ignoring the initial positive baseline correlation between amyloid-β pathology and intrinsic connectivity, previous studies therefore may have underestimated the resulting ‘negative’ impact of amyloid-β on connectivity. As a consequence, connectivity reductions in amyloid-β-positive cohorts may only be robust enough to be noticeable once the accumulation is already substantial. This argument also applies to different brain areas: high lifetime connectivity in region X (compared to region Y) increases amyloid-β plaque accumulation, leading to a larger connectivity reduction (that is nevertheless masked by higher baseline connectivity). On average, the DMN seems to show the highest amyloid-β aggregation, making it the network in which amyloid-β-related connectivity reduction is easiest to detect. This could therefore lead to an overemphasis of this network in studying Alzheimer’s disease, even though other networks are also affected.

Here, we addressed both of these issues using a novel methodological approach that for the first time attempts to disentangle the positive and negative relationship between amyloid-β and intrinsic connectivity within the same patient cohort. To this end, we used multimodal imaging that estimated regional plaque load [via Pittsburgh Compound B (PiB)-PET] and intrinsic connectivity (via resting-state functional MRI) in patients with prodromal Alzheimer’s disease harbouring significant amyloid-β-plaque pathology and in healthy controls. The use of within-patient statistics was central to our approach for two reasons: for one, it would otherwise be impossible to disentangle negative and positive relationships. Additionally, it allowed us to confirm previous studies done in animal models (e.g. Bero *et al.*, 2011, 2012) or using intrinsic connectivity estimates from healthy controls (e.g. Buckner *et al.*, 2009, Seeley *et al.*, 2009, Zhou *et al.*, 2012) that may not necessarily replicate in patients (Huang and Mucke, 2012). In addition, we recruited a control group of healthy persons without significant amyloid-β-plaque pathology. Although our within-subject approach does not strictly require a control group, we wanted to ensure that any effects do in fact depend on amyloid-β pathology. In the patient group, we found that the distributions of amyloid-β plaques and intrinsic networks within a number of heteromodal fronto-parietal networks are positively correlated, confirming a relationship that had previously been established only for the DMN. Critically, by taking this positive relationship into account, we found that our approach led to a substantial increase in the sensitivity of detecting the negative impact of amyloid-β pathology on intrinsic connectivity, compared to conventional group comparisons of connectivity.

## Methods and materials

### Participants

Twenty-four patients (10 female, age range 50–83 years) diagnosed with prodromal Alzheimer’s disease (using standard diagnostic criteria, see below) and 16 healthy controls (nine female, age range 57–75 years) participated in the study (Table 1). All participants provided informed consent in accordance with the Human Research Committee guidelines of the Klinikum Rechts der Isar, Technische Universität, München. Patients were recruited from the Memory Clinic of the Department of Psychiatry, and controls by word-of-mouth advertising. Examination of every participant included medical history, neurological examination, informant interview (Morris, 1993), neuropsychological assessment by the neuropsychological assessment battery of the Consortium to Establish a Registry for Alzheimer’s disease (CERAD, Morris *et al.*, 1989), structural MRI and PiB-PET. Prodromal Alzheimer’s disease has recently been defined by the coincidence of both mild cognitive impairment and the presence of at least one of five supportive biological signs for Alzheimer’s disease, such as medial temporal lobe atrophy or significant PiB uptake (Dubois *et al.*, 2007). Criteria for mild cognitive impairment include reported and neuropsychologically assessed cognitive impairments, largely intact activities of daily living, and excluded dementia (Gauthier *et al.*, 2006). Patients in our study met criteria for mild cognitive impairment and demonstrated significant cortical PiB-uptake (i.e. they were PiB-positive). We used a cut-off for ‘high’ or ‘low’ neocortical standardized uptake value ratios of 1.15, consistent with cut-off values used in previous PiB-PET studies (Drzezga *et al.*, 2011). Patients with high PiB binding (i.e. standardized uptake ratio ≥1.15) were classified as PiB-positive and those with standardized uptake ratio <1.15 were classified as PiB-negative, which was an inclusion criterion for healthy control subjects (see Supplementary material and Supplementary Fig. 4). Standardized PiB-uptake is measured for a pre-established large cortical volume of interest including lateral prefrontal, parietal, and temporal areas and the retrosplenial cortex (Hedden *et al.*, 2009; Drzezga *et al.*, 2011). Exclusion criteria for entry into the study were other neurological, psychiatric or systemic diseases (e.g. stroke, depression, alcoholism), or clinically remarkable structural MRI (e.g. stroke lesions) potentially related to cognitive impairment. Fifteen patients and eight healthy control subjects were treated for hypertension (beta-blockers, ACE-inhibitors, and calcium channel blockers), and seven patients and five healthy control subjects were treated for hypercholesterolaemia (statins). Two patients had diabetes mellitus, four patients received antidepressant medication (mirtazapine, citalopram), and no patient received cholinesterase inhibitors.

**Table 1:** Demographic and clinical-neuropsychological data

	Groups	
	Patients	Controls
*n*	23	12
Age	69.3 (7.4)	63.8 (5.15)*
Gender (F/M)	9/14	9/5
CDR global	0.5 (0)	0 (0)*
CDR-SB	1.6 (0.5)	0 (0)*
CERAD total	66.3 (10.8)	88.1 (6.8)*

CDR = Clinical Dementia Rating; CDR-SB = CDR sum of boxes; CERAD = neuropsychological assessment battery of the Consortium to Establish a Registry for Alzheimer’s disease; CERAD-total = summary of CERAD subtests; group comparisons: χ^2^ (gender), two-sample *t*-test (age, CDR global, CDR-SB, CERAD-total).

*Significant group difference with *P < *0.05.

All participants underwent both MRI and PET imaging sessions. The MRI session included structural MRI and resting-state functional MRI acquisition. PET and MRI sessions were conducted within 3.7 (±2.5) months for patients, and within 8 (±3.1) months for healthy controls. One patient and four control participants were excluded from further analysis because of corrupted PET data, resulting in all analyses being conducted on 23 patients and 12 healthy control participants.

### Pittsburgh Compound B-positron emission tomography imaging and data analysis

PET-imaging with *N*-methyl-^11^C-2-(4-methylaminophenyl)-6-hydroxybenzothiazole (Pittsburgh Compound B) and data analysis followed standard protocols as described in a previous study (Mosconi *et al.*, 2008). All participants were injected with 370 MBq ^11^C-PiB at rest before entering the scanner 30 min later. Forty minutes post-injection, three 10-min frames of data acquisition were started and later summed into a single frame (40–70 min). Acquisition was performed using a Siemens ECAT HR+ PET scanner (CTI) in 3D mode and a transmission scan was carried out subsequently to allow for later attenuation correction.

The first step of imaging data analysis consisted of image reconstruction, correction of dead time, scatter and attenuation. Statistical parametric mapping software (SPM 5, Wellcome Department of Cognitive Neurology, London, UK) was used for image realignment, transformation into standard stereotactic space (MNI PET template), smoothing and statistical analysis (Mosconi *et al.*, 2008). For the spatial transformation of PiB data, standardized uptake value images (40–70 min post injection) were co-registered to each individual’s volumetric MRI and then automatically spatially normalized to the MNI-template in SPM5 using warping parameters derived from previous individual structural MRI normalization (Mosconi *et al.*, 2008). For each subject, all voxel values were normalized to the cerebellar vermis. Additionally, images were smoothed (Gaussian kernel of 10 mm × 10 mm × 10 mm) for the group comparisons. For the regression and searchlight analyses, we used unsmoothed PET images. Whole brain voxel-wise group comparisons (two-sample *t*-test) were performed with a threshold of *P < *0.0001 uncorrected and k = 100.

### Magnetic resonance imaging data acquisition and analysis

MRI was performed on a 3 T whole body MR scanner (Achieva) using an 8-channel phased-array head coil. For co-registration, T_1_-weighted anatomical data were obtained from each participant using a MPRAGE sequence (echo time = 4 ms, repetition time = 9 ms, inversion time = 100 ms, flip angle = 5°, field of view = 240 × 240 mm^2^, matrix = 240 × 240, 170 slices, voxel size = 1 × 1 × 1 mm^3^). Functional MRI data were collected using a gradient echo echo planar imaging sequence (echo time = 35 ms, repetition time = 2000 ms, flip angle = 82°, field of view = 220 × 220 mm^2^, matrix = 80 × 80, 32 slices, slice thickness = 4 mm, and 0 mm interslice gap, 300 volumes).

For each participant the first three functional scans of each functional MRI session were discarded because of magnetization effects. SPM5 (Wellcome Department of Cognitive Neurology, London) was used for data preprocessing. First, we used affine coregistration (to the first image) to motion-correct the resting-state functional MRI data. We observed no excessive head motion (i.e. cumulative translation or rotation >3 mm or 3° and mean point-to-point translation or rotation >0.15 mm or 0.1°). Framewise displacement (Power *et al.*, 2012) or the root-mean-square of translational parameters (Van Dijk *et al.*, 2012) were not different between groups (*P > *0.05, two-sample *t*-tests). Further, groups yielded no significant differences in signal-to-noise ratio of functional MRI data (*P* > 0.05). The high-resolution structural image was coregistered to the mean functional MRI image (using affine registration), and normalized to a template in the stereotactic space of the Montreal Neurological Institute (MNI) with the ‘segment’ function (SPM5), which uses an iterative combination of non-linear registration and cortical segmentation (Ashburner and Friston, 2005). Normalization was then applied to the functional images before smoothing with an 8 × 8 × 8 mm^3^ Gaussian kernel.

As described previously (Sorg *et al.*, 2013), the preprocessed data were decomposed into spatially independent components reflecting intrinsic networks in a group-independent component analysis framework (Calhoun *et al.*, 2001), which is implemented in the GIFT software (http://icatb.sourceforge.net). Our independent component analysis approach consisted of a series of well-established analysis steps (Kiviniemi *et al.*, 2003; Erhardt *et al.*, 2011). We estimated data dimensionality using a minimum description length criterion, which gave an estimate of 35 components (the mean of all individual dimensionality estimates). Data from all participants were temporally concatenated into one data set. The estimation of independent component analysis across both groups was in line with previous research (e.g. Filippini *et al.*, 2009) and ensured a better correspondence of network maps between groups. Concatenated data were reduced by two-step principal component analysis to reduce computational burden. Principal component analysis was followed by independent component analysis with the infomax-algorithm. We ran 40 independent component analyses (ICASSO) to ensure stability of the estimated components. This results in a set of averaged group components, which are then back-reconstructed into single-subject space. For each subject, each component was represented as a combination of a network time course and a spatial map of *z*-scores. The *z*-map reflects the component’s functional connectivity pattern (i.e. the mixing weights) across the brain. Voxels whose time courses are highly correlated with the component time course receive high connectivity *z*-scores, whereas voxels that are not part of the network have *z*-scores near 0.

Following previous findings of aberrant medial and lateral heteromodal frontoparietal networks in early Alzheimer’s disease (Sorg *et al.*, 2007, 2009; Neufang *et al.*, 2011; Agosta *et al.*, 2012), the DMN and so-called attentional networks were of *a priori* interest (Allen *et al.*, 2011). To automatically select networks of interest, we applied multiple spatial regression analyses of the 35 independent components on masks derived from a previous study (Allen *et al.*, 2011): the anterior and posterior DMN [aDMN IC 25, pDMN IC 53 of Allen *et al.* (2011)], attentional networks (ATN; right ATN IC 60, left ATN IC 34, dorsal ATN IC 72, salience network SN IC 55), and, as a control, a network around the primary auditory cortex (pAN; IC 17). Masks were generated with the WFU-Pickatlas (http://www.fmri.wfubmc.edu/).

To evaluate statistically the spatial z-maps of selected components, we calculated voxel-wise one-sample *t-*tests on participants’ reconstructed spatial maps for each group, using SPM5 (*P < *0.05, family wise error (FWE) corrected at cluster level, voxel-level height threshold *P < *0.001). To analyse group differences, corresponding spatial z-maps were entered into two-sample *t*-tests, restricted to appropriate one-sample *t*-test masks (*P < *0.01 uncorrected, calculated prior to group comparison) across all subjects (*P < *0.05, FWE cluster level, voxel-level height threshold *P < *0.001). As a more conservative test of group differences, for each subject and network we also identified all network voxels (as voxels with a connectivity z-score >1) and calculated the median (log-transformed) z-score across the entire network. Resulting network connectivity scores were then submitted to two-way mixed-effects ANOVA with factors group and network.

### Multimodal analyses

**Figure 1:**
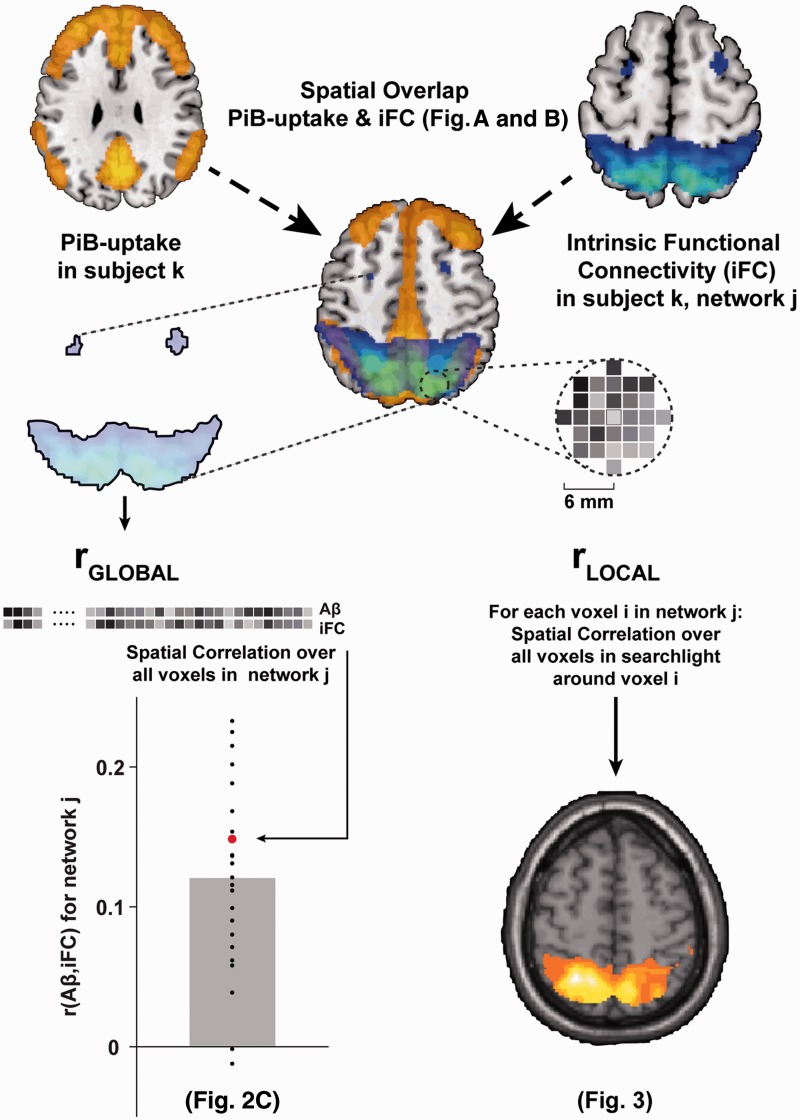
Overview of analysis approach. For each individual, voxel-wise PiB-uptake was determined as a measure of amyloid plaque density. For each intrinsic network j (as determined by resting-state functional MRI and independent component analysis independent component analysis) in each individual k, we identified the voxels belonging to that network (*top right*) and extracted the intrinsic functional connectivity (iFC) values of those (∼10 000) voxels. We also extracted that individual’s PiB-uptake values for the same voxels. To determine average plaque load in each network, we then calculated the median PiB-uptake (shown in Fig. 2A and B). We then calculated global and local correlations between both modalities across the entire network and confined to neighbouring voxels. To determine the global spatial correlation between intrinsic functional connectivity and PiB-uptake, we calculated the Pearson correlation coefficient across all selected voxels in the network (r_GLOBAL_, *bottom left* and Fig. 2C). After correcting for r_GLOBAL_ (via orthogonalization, see ‘Materials and methods’ section), we then used a searchlight approach to calculate local spatial correlation in the neighborhood surrounding each voxel in the network (r_LOCAL_, *bottom right* and Fig. 3). Aβ = amyloid-β.

Figure 1 gives an overview of our multimodal analysis approach. In a first step, we analysed the extent of PiB-uptake in the different intrinsic networks. For each subject and network, we identified all voxels belonging to the network (i.e. with a *z*-scored connectivity weight of ≥1 in the individual back-projected maps). We then calculated (for each subject) the median PiB-uptake across all voxels in the network. To control for the potential effects of network-wise grey matter density, age or gender, we then regressed these variables out of the median PiB data (Supplementary material, Supplementary Fig. 2 and Supplementary Table 8). For each network and separately for each group, we fit to the median PiB values a linear model consisting of three regressors: median grey matter density (in that network), age and gender. None of the three covariates had a notable effect on PiB-uptake (or on r_GLOBAL_ or r_LOCAL_, see below). We subtracted model-predicted PiB values from the real data to obtain residuals that were independent of the three covariates. These residual PiB values were evaluated with a mixed between- and within-subject effects ANOVA (with factors group (patient/control) and network) and *post hoc* two-sample *t*-tests.

Next, we tested for spatial correlations between PiB-uptake and network connectivity across the entire network (r_GLOBAL_). Again, for each subject and network we identified network voxels as all voxels with a connectivity z-score >1. For all voxels, we extracted PiB-uptake and connectivity values. We log-transformed connectivity values to reduce the skew in their distribution, which was induced by including only values >1 (although analyses using untransformed connectivity values gave qualitatively and statistically comparable results). For each subject and network, we calculated the Pearson correlation coefficient between PiB-uptake and connectivity *z*-values, and Fisher-transformed the resulting r-values. The resulting r_GLOBAL_ values were again (as with PiB-uptake, above) corrected for grey matter density, age, and gender, and the residuals were submitted to a mixed-effects ANOVA and *post hoc t*-tests (as above).

To investigate the local impact of amyloid-β pathology on intrinsic connectivity, we used local spatial correlations between PiB-uptake and connectivity (r_LOCAL_) via a searchlight approach. Searchlight approaches (Kriegeskorte *et al.*, 2006) are multivariate analysis methods that step through all voxels of interest in sequence (in our case, all voxels in a given network) and examine voxel values in a ‘searchlight’ region of interest surrounding the current voxel. The measure of interest is then recorded in that voxel, resulting in a spatial map of local multivariate measures. We again identified the same set of voxels for each subject and network. As for this analysis we were explicitly interested in local variability between PiB-uptake and connectivity that was not already accounted for by the global positive correlation (r_GLOBAL_), we first decorrelated PiB-uptake and connectivity values across the whole network. To this end (see Supplementary Fig. 3), we used Gram-Schmidt orthogonalization to decorrelate PiB uptake with respect to connectivity *z*-values across the entire network (ensuring a network correlation of 0). This approach has been used to remove zero-lag correlations when connectivity is calculated in magnetoencephalographic data (see Brookes *et al.*, 2011; Hipp *et al.*, 2012). As orthogonalization is an asymmetric operation (modifying one vector while leaving the other unchanged), we repeated the orthogonalization and subsequent searchlight analysis, but this time decorrelating connectivity *z*-values with respect to PiB-uptake. The searchlight results of the two analyses were then averaged.

Next, for each voxel in the network, we identified all voxels in its immediate neighbourhood (i.e. within a 6-mm radius, typically ∼100 voxels). To avoid unreliable estimates of r_LOCAL_ at network boundaries, all voxels with less than 25 voxels in their neighborhood were skipped. Then we calculated the Pearson correlation between PiB-uptake and connectivity *z*-values in that neighbourhood, and recorded the (Fisher-transformed) correlation coefficient in the neighbourhood’s central voxel, resulting in a spatial map of r_LOCAL_ values for each subject and network. For each group and network, we submitted these maps to one-sample *t*-tests using SPM. In addition, for each subject and network we extracted the median r_LOCAL_ value across all voxels, and submitted the corrected values (after regressing out covariates of no interest) to a mixed-effects ANOVA and *post hoc t*-tests (as above).

## Results

### Reduced intrinsic connectivity in the default mode and right attention networks in patients with prodromal Alzheimer’s disease

**Figure 2:**
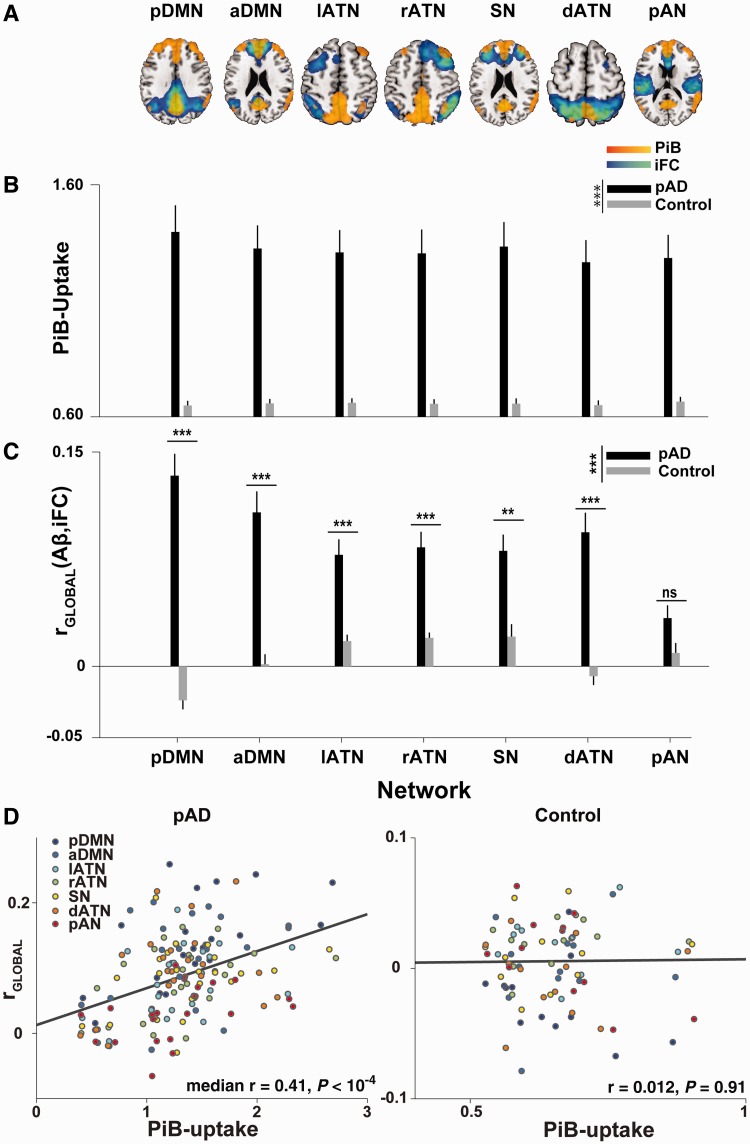
Amyloid-β deposition is increased in a number of heteromodal networks and correlates with intrinsic functional connectivity. (**A**) Each image shows a group-level intrinsic functional connectivity (iFC) network map (cold colours) at a FWE-corrected threshold of *P < *0.05, superimposed on the group-level difference map in amyloid-β deposition (warm colors), thresholded at *P < *0.0001, minimum cluster extent of 100 voxels. The pattern of amyloid-β (Aβ) deposition overlaps noticeably with a number of fronto-parietal networks (lATN = left lateral frontoparietal ATN; rATN = right frontoparietal ATN; dATN = posterior parietal dorsal ATN; SN = salience network) besides the DMN [split into a posterior (pDMN) and anterior (aDMN) component], and even with regions of primary sensory cortex, such as the primary auditory network (pAN). Notably, in some networks amyloid-β plaque deposition appears limited to network hubs (as in the left ATN and dorsal ATN, where frontal network regions lack significant amyloid-β burden at the group level). (**B**) Patients showed significantly increased PiB-uptake in all tested networks [main effect of group across seven networks, *F*(1,33) = 22.11, *P < *10^−4^]. There was also a main effect of network that interacted with group [main effect of network for patient group, *F*(6,132) = 5.37, *P < *10^−4^], indicating that there is a gradient of amyloid-β deposition across networks in patients. (**C**) A number of networks in the patient group exhibited a positive network-wide correlation between voxel-wise intrinsic functional connectivity and voxel-wise PiB-uptake (i.e. r_GLOBAL_), indicating that voxels in the network core (i.e. voxels with high intrinsic functional connectivity) were burdened with higher amyloid-β deposition than the network periphery (voxels with low intrinsic functional connectivity). This correlation was modest in magnitude but consistently positive [main effect of group, *F*(1,33) = 47.19, *P < *10^−7^, *t*-tests of Fisher-transformed Pearson correlation coefficients against control group, all *t*(33) > 3.61, Bonferroni-corrected *P < *0.01, except for the auditory network, *t*(33) = 1.87, non-significant]. Correlations in the control group were negligible. There was a gradient of correlation strength across networks as the six default and attention networks showed a stronger correlation than the posterior auditory network. Also, the amyloid-β–intrinsic functional connectivity correlation was stronger in the posterior DMN than in any of the other networks except the anterior DMN. Error bars indicate standard error of the mean. ***P < 0.001, **P < 0.01, *P < 0.05, all Bonferroni-corrected, ns = not significant. (**D**) Median PiB-uptake is positively correlated with r_GLOBAL_ in patients (*left*), but not in controls (*right*). Each point in the scatter plot represents one network in one individual, with network identity denoted by colour. The regression lines are derived from a linear fit across all individuals and networks in each group. Nevertheless, correlations were also performed separately for each network.

Using independent component analysis of resting-state functional MRI data, we identified six fronto-parietal heteromodal networks (Fig. 2 and Supplementary Fig. 1). These networks included the posterior and anterior DMN, the left, right and dorsal attention networks (all covering the lateral parietal and prefrontal cortex), and the fronto-limbic salience network, covering the insula and anterior cingulate cortex. As a control, we also selected a primary sensory network (the primary auditory network). Group comparisons revealed regionally reduced *z*-scored connectivity values in the prodromal Alzheimer’s disease group (two-sample *t*-tests, *P < *0.05 FWE cluster corrected for multiple comparisons) only in posterior parietal regions of the posterior DMN and right attention networks, replicating previous results (e.g. Sorg *et al.*, 2007; see Supplementary Fig. 1).

### Pittsburgh Compound B uptake in prodromal Alzheimer’s disease extends to lateral fronto-parietal attention network

In patients with prodromal Alzheimer’s disease, we found significantly increased PiB-uptake (compared to controls) in a large number of cortical regions, including areas of the DMN, but extending to the lateral parietal and frontal lobes (Fig. 2A), corresponding well with characteristic distribution patterns previously described in patients with Alzheimer’s disease and mild cognitive impairment (Drzezga *et al.*, 2011).

Next, we tested quantitatively how PiB-uptake overlaps with intrinsic connectivity networks. We extracted the median PiB-uptake in each of these networks. After correcting for grey matter density, age and gender, we compared the network-wise PiB-uptake between prodromal Alzheimer’s disease and control subjects (with ANOVA and two-sample *t*-tests). As already suggested by visual inspection (Fig. 2A), the patient group had significantly increased PiB-uptake [Fig. 2B: main effect of group across seven networks, *F*(1,33) = 23.94, *P < *10^−4^]. Importantly, patients showed a gradient of plaque deposition across networks [*F*(6,132) = 3.83, *P = *0.001], with the highest PiB-uptake in the posterior DMN [significantly increased compared to left ATN and right ATN, all *t*(22) > 3.12, corrected *P < *0.035], and strong trends compared to anterior DMN [*t*(22) = 2.95, corrected *P = *0.052], and to posterior auditory cortex network [*t*(22) = 2.77, corrected *P = *0.078].

### Spatial patterns of Pittsburgh compound B uptake correlate positively with those of intrinsic connectivity in default mode and attention networks

After confirming the presence of amyloid-β-pathology in a number of fronto-parietal networks, we examined the relationship between amyloid-β-pathology and intrinsic connectivity. For each network and subject, we calculated the spatial correlation coefficient between PiB-uptake and connectivity-reflecting *z*-values over all network voxels, i.e. r_GLOBALiFC_ [amyloid-β, intrinsic functional connectivity (iFC)] (see Supplementary Fig. 5 for a representative scatterplot of voxel-wise PiB-uptake against connectivity in one patient). For each network (except the auditory network, posterior AN), we found a modest but robust positive correlation between amyloid-β and connectivity in patients [Fig. 2C, main effect of group, *P < *10^−7^, mean correlation coefficients ranging from 0.134 (posterior DMN) to 0.078 (left ATN), corrected *P < *0.002 in all networks except for the auditory network, mean r = 0.034, *t*(33) = 2.26, non-significant]. Correlations in the control group did not deviate from 0 (mean r < ±0.024, *P* = 0.136).

In analogy to the observed gradient in PiB-uptake across networks (Fig. 2A and B), we found a gradient of r_GLOBAL_ across networks [*F*(6,132) = 10.72, *P < *10^−7^] as the six default and attention networks showed a stronger r_GLOBAL_ than the auditory network [all *t*(22) > 3.45, corrected *P < *0.014]. r_GLOBAL_ was also stronger in the pDMN than in any of the other networks except the aDMN [all *t*(22) > 3.31, corrected *P < *0.019, paired *t*-test between posterior DMN and anterior DMN, *t*(22) = 1.81, uncorrected *P = *0.084]. Finally, average PiB-uptake was positively correlated with r_GLOBAL_ in patients, but not in controls (Fig. 2D, posterior DMN in patients, r = 0.64, *P < *0.01, median r = 0.47 for patients, median r = 0.06 for control subjects). In contrast, r_GLOBAL_ did not correlate with mean intrinsic connectivity in any network of either group (all uncorrected *P* > 0.12), indicating that the correlation was mainly driven by variability in PiB-uptake.

### In network cores of higher connectivity, amyloid-β pathology has a negative impact on functional connectivity

Next we focused on the concomitant ‘negative’ impact of amyloid-β load in local network regions: specifically, after accounting for the variability in amyloid-β-pathology that is attributable to r_GLOBAL_, the neurotoxic effects of higher pathology should lead to a ‘relative decrease’ in intrinsic connectivity, especially in network cores with high connectivity (where amyloid-β pathology presumably has been accumulating for longer; Bero *et al.*, 2012). To account for the diluting effects of r_GLOBAL_, we regressed out the impact of intrinsic connectivity on plaque deposition at the whole-network level and used a searchlight approach to calculate the local spatial correlation of the residuals around each voxel (see Fig. 1); we called this measure r_LOCAL_(Aβ,iFC) to indicate that here we calculated correlations in small (6-mm radius), homogeneous neighbourhoods around each voxel (in every network and subject), yielding a local measure of amyloid-β pathology impact that could potentially vary ‘within’ networks.

**Figure 3:**
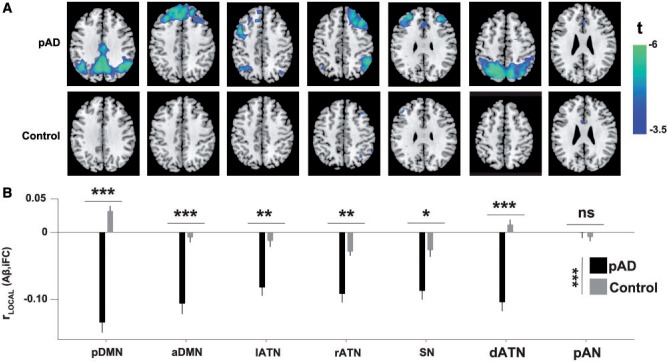
Local negative impact of amyloid-β on intrinsic connectivity. (**A**) Searchlight analysis demonstrated negative correlations between amyloid-β plaques (Aβ) and intrinsic functional connectivity (iFC) in small neighbourhoods with generally homogeneous connectivity strength. After accounting for the impact of intrinsic functional connectivity on PiB-uptake across an entire network (i.e. r_GLOBAL_ described in Figs 1B and 2) through orthogonalization, we calculated at each network voxel the local amyloid-β–intrinsic functional connectivity (Aβ,iFC) correlation r_LOCAL_ across surrounding voxels in a 6-mm radius. For each group, we submitted the subject-level maps of correlation coefficients to *t*-tests, using FWE to correct for multiple comparisons. In the prodromal AD group (*top*), all networks except for the auditory network (pAN) showed significant negative r_LOCAL_ across large parts of the networks, focusing on network hubs. There were no such correlations in the control group (*bottom*). Colour scale indicates *t*-statistics at the group level. A *t*-value of 3.5 corresponds to *P = *0.001, uncorrected. (**B**) In patients, median r_LOCAL_ values were significantly below 0 for all networks except the auditory network. The control group showed no significant correlations. Hence, direct comparisons between patients and controls revealed a strong main effect of group [*F*(1,33) = 43.25, *P < *10^−6^, all *t*(33) <−3.17, corrected *P < *0.025], with the exception of the auditory network, which showed no difference in median r_LOCAL_ [*t*(33) = 0.54, ns]. Error bars indicate standard error of the mean. a/pDMN = anterior/posterior DMN; l/r/dATN = left/right/dorsal ATN; SN = salience network.

In patients (Fig. 3A), all networks except for the pAN showed significant negative r_LOCAL_ across large parts of the network, peaking in network cores. For example, in the dorsal attention network (Fig. 3A), r_LOCAL_ was significantly below zero in the superior parietal core of the network, but not strongly negative in peripheral regions such as the frontal eye fields (compare to network distribution in Fig. 2A or Supplementary Fig. 1). r_LOCAL_ did not deviate from 0 in the control group (Fig. 3A). In addition to comparing voxel-wise maps, we also examined the median r_LOCAL_ values across all voxels in a network (Fig. 3B). r_LOCAL_ was significantly below 0 for all networks [all *t*(22) < −6.73, corrected *P < *10^−5^] except the auditory network [*t*(22) = −0.04]. Direct comparisons between patients and controls revealed a strong main effect of group [*F*(1,33) = 44.21, *P < *10^−6^, all *t*(33) < −3.65, corrected *P < *0.006], with the exception of the posterior auditory network, which showed no difference in median r_LOCAL_ [*t*(33) = 0.68, non-significant]. As suggested by the spatial distribution of r_LOCAL_ in Fig. 3A, we also found a correlation between PiB-uptake and r_LOCAL_. In other words, local regions with higher amyloid-β pathology overall (where plaques have presumably been accumulating for longer and may have had a stronger connectivity-reducing effect on neurons) also exhibit a stronger negative impact of amyloid-β pathology on connectivity (Supplementary Fig. 3). Together, our results show that after accounting for the globally positive relationship between amyloid-β and intrinsic connectivity, local neighbourhoods exhibit a strong negative influence of plaques on connectivity, even in networks where typical estimates do not yet indicate connectivity reductions (see Supplementary Fig. 1 and Supplementary Tables 1–7 for direct comparisons of connectivity between groups).

### Control analyses

Finally, we tested whether either differences in group size or any methodological steps (such as orthogonalization of PiB-uptake and intrinsic connectivity) could have influenced our results. To control for the difference in group size, we used a subsampling approach that repeatedly selects a random subgroup of patients, matched in size with the control group, and re-computed statistics for this smaller, matched data set (Supplementary material and Supplementary Table 9). All relevant comparisons remained significant (with the exception of r_GLOBAL_ and r_LOCAL_ in the salience network and left ATN, and r_LOCAL_ in right ATN, which nevertheless are highly significant at an uncorrected threshold). Further, control analyses (e.g. concerning orthogonalization, see Supplementary material and Supplementary Fig. 3) showed that negative r_LOCAL_ does not depend on specific steps of our analysis approach.

## Discussion

In patients with prodromal Alzheimer’s disease, spatial correlations revealed two distinct effects of PiB-uptake on intrinsic connectivity within individual persons and in several heteromodal intrinsic networks. First, at the global network level, patterns of amyloid-β plaques correspond spatially with patterns of connectivity, with the highest correlation in networks carrying the highest plaque load. Second, at the local network level, plaques are negatively associated with connectivity, especially in regions of high connectivity. These results extend previous findings by demonstrating the negative impact of amyloid-β pathology on intrinsic connectivity beyond the DMN and by showing a general pattern of correlations between plaques and connectivity within and across intrinsic networks.

### In heteromodal intrinsic networks, patterns of plaques correspond with patterns of intrinsic connectivity

In several heteromodal fronto-parietal networks, including the DMN and lateral attention networks, we found within-patient spatial correlations between amyloid plaque distributions and intrinsic connectivity (Fig. 2C). We had anticipated this outcome, based on previous findings (at the group level) of positive correlations centred on the DMN (Buckner *et al.*, 2009). This finding was specific for heteromodal networks, as we saw no effect in the primary auditory network (Fig. 2C). The dissociation between heteromodal and primary networks is in line with findings that primary sensory and sensorimotor regions are relatively spared in earlier stages of Alzheimer’s disease (Braak and Braak, 1991). In all of the networks exhibiting a significant spatial correlation, patients with prodromal Alzheimer’s disease had significant plaque load (Fig. 2A and B), with the highest load in the posterior DMN. Generally, the correlation between plaques and connectivity was higher in networks of higher median plaque load (Fig. 2C and D). Inclusion of control variables confirmed that these findings were not influenced by grey matter atrophy, age, or gender, and that they were specific to heteromodal networks, since we did not find comparable results in the primary auditory network (Fig. 2).

Our observations are in line with recent results by Drzezga *et al.* (2011), who found that patterns of both plaques and hubness (i.e. the average connectivity of any one region or voxel to the rest of the brain, irrespective of network boundaries; see also Buckner *et al.*, 2005, 2009) overlap in patients with significant plaque load. These studies focused on effects in the DMN, as their methodological approach did not differentiate between networks and therefore emphasized areas with the highest hubness, which tend to be in the DMN. Here, we extended these studies by confirming that the plaque/connectivity correlation exists at the level of individual intrinsic heteromodal networks beyond the DMN, and by showing that it does not rely on network-unspecific hubness. Our finding provides strong evidence for the previously stated hypothesis (Jagust and Mormino, 2011) that high levels of connectivity in heteromodal areas are associated with increased levels of amyloid-β pathology, potentially due to nodal stress incurred by a lifetime of increased intrinsic activity (Bero *et al.*, 2011).

### Accounting for network level plaque–connectivity correlations reveals the widely distributed negative impact of amyloid-β pathology on intrinsic connectivity

After accounting for global plaque-connectivity correlations across a network, we found negative correlations (r_LOCAL_, in small neighbourhoods of ∼100 voxels) between the local plaque distribution and intrinsic connectivity in several heteromodal networks, particularly in regions of high intrinsic connectivity (Fig. 3). These negative correlations were stronger in networks of high plaque load (Supplementary Fig. 3), suggesting a detrimental impact of amyloid-β pathology on intrinsic connectivity. This finding is in line with results of previous studies, which found intersubject correlations between amyloid-β load and intrinsic connectivity in the DMN in individuals with significant plaque pathology (Hedden *et al.*, 2009; Sheline *et al.*, 2010; Mormino *et al.*, 2011).

Our results extend these findings in two ways. First, we showed the negative impact of pathology on connectivity within single patients, instead of across patients. Second, we found significant results in heteromodal networks beyond the DMN. With respect to the latter point, our approach appears to be more sensitive than conventional methods used to detect connectivity reductions (Supplementary Fig. 1). Although our spatial correlation approach revealed strong and widespread effects in several networks, simple group comparisons of voxel-wise connectivity found reduced connectivity only in the posterior DMN and the right attention network (Supplementary Fig. 1). Control analyses (Supplementary Fig. 3 and Supplementary material) confirmed that removing the globally positive whole-network baseline correlation between plaques and connectivity is critical for this increase in sensitivity. Approaches that neglect baseline plaque/connectivity correlations potentially underestimate disease effects on intrinsic connectivity. Given that significant PiB-uptake is measurable in the DMN even in early preclinical stages of Alzheimer’s disease (Jack *et al.*, 2010; Bateman *et al.*, 2012), where conventional measures of connectivity do not report robust connectivity reductions, the method presented here may help detect early, subtle reductions in connectivity. Future studies or re-analyses are necessary to test these suggestions.

### Gradients of plaques along intrinsic connectivity across and within networks furnish an extended network degeneration model for Alzheimer’s disease

**Figure 4:**
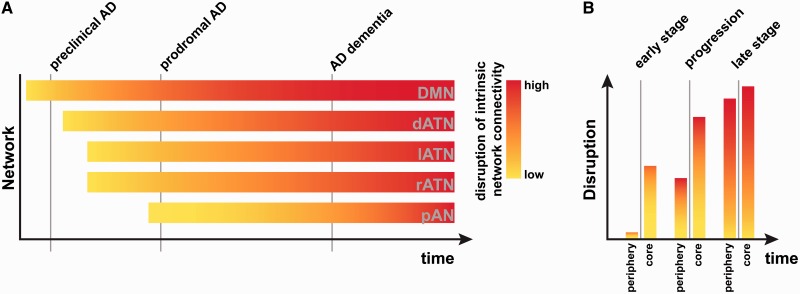
Proposed model of graded network degeneration. Graded network degeneration hypothesis of amyloid-β propagation in Alzheimer’s disease (AD). *Left*: In spite of a predominant focus on amyoid-β pathology accumulation and spread along intrinsic connectivity in the DMN [*top* bar, with colour indicating the (putatively amyloid-related) connectivity decrease over time], amyloid-β plaques are also found in other cortical regions that typically have lower resting-state metabolism and connectivity than the DMN (e.g. left and right ATNs). In an average person with prodromal Alzheimer’s disease, amyloid-β pathology deposition and connectivity-based spread will start in the DMN, followed in close succession by a number of other heteromodal networks, leading to a cross-network gradient (i.e. along the vertical grey lines cutting across networks). We propose that the same principle is at work ‘within’ networks (*right*), where the network core will accumulate amyloid-β and show signs of disruption sooner than areas in the periphery.

Our results suggest a view of Alzheimer’s disease that goes beyond previous models that focus on both the DMN and its associated amyloid-β-plaque accumulation while ignoring other intrinsic networks and the complex relationship between connectivity-mediated amyloid-β-increases on a network scale and local amyloid-β-mediated connectivity decreases. Although we confirmed that PiB-uptake and its effects on intrinsic connectivity were strongest in the posterior DMN (as measured both by r_GLOBAL_ and r_LOCAL_; Figs 2 and 3), we focused on the well-known but somewhat neglected finding that, even in prodromal Alzheimer’s disease, amyloid-β-plaques accumulate outside the DMN (Fig. 2A and B). We found that PiB-uptake was higher in the network core, where network connectivity (and, presumably, neural activity and metabolism) is higher than in the periphery. A speculative model (Fig. 4) summarizes our results and illustrates the graded (but temporally overlapping) effects of amyloid-β-pathology across networks (that are strongest in the posterior DMN, but robust in other networks as well).

We propose that the within-network effects of amyloid-β-pathology will be similar in all affected networks (but possibly shifted in time depending on how early amyloid-β plaques aggregate in a particular network).

One speculative explanation for our findings is that in general, plaque accumulation follows connectivity (leading to the positive correlation). In addition, wherever plaque load is high, plaques have been accumulating longer, and have exerted a negative influence on local intrinsic activity and connectivity for longer. Viewed in this light, our findings are in line with animal and computational studies linking amyloid-β pathology to increased connectivity in early stages (i.e. increased r_GLOBAL_) and decreased connectivity (i.e. negative r_LOCAL_) in later stages of Alzheimer’s disease (Bero *et al.*, 2012; de Haan *et al.*, 2012). In contrast with many previous models that describe the temporal staging of Alzheimer’s disease as a fixed progression, our model acknowledges that some brain networks (and areas within those networks) will be affected by pathology before others. A hub within a network that was affected early on might already show neural effects reminiscent of late Alzheimer’s disease, while at the same time another network might show only the neural effects associated with early Alzheimer’s disease.

### Importance of within-patient spatial correlations between Pittsburgh compound B uptake and connectivity scores

In contrast to previous studies, which tested amyloid-β-propagation models in healthy controls and compared them with patient atrophy data (Buckner *et al.*, 2009; Seeley *et al.*, 2009; Raj *et al.*, 2012; Zhou *et al.*, 2012), or which correlated amyloid-β plaques with DMN connectivity across subjects (Drzezga *et al.*, 2011), here we developed a methodological frame that allowed us to analyse the within-subject relationship between amyloid-β pathology and functional connectivity. Although the importance of animal models and connectivity measures in healthy subjects is unquestionable, it is essential to transfer findings from disease models to patients. For instance, mouse models show clear differences in amyloid-β aggregation and clearance compared with patients, and the effect of amyloid-β plaques on cognition is not necessarily comparable (Hall and Roberson, 2012; Huang and Mucke, 2012). By looking at within-subject-correlations, we can reduce the possibility that third variables (such as disease state in general) are mediating the correlation between amyloid-β and intrinsic connectivity. More importantly, it allows us to estimate the impact amyloid-β has had on functional connectivity at the single subject and single-network level. This increase in sensitivity compared to conventional measures of intrinsic connectivity may facilitate earlier detection of the functional impact of amyloid accumulation in incipient disease. In future studies, it may additionally help to differentiate patients with prodromal Alzheimer’s disease from patients whose mild cognitive impairment was caused by another form of neurodegeneration. The correct differential diagnosis is an essential component of developing more sensitive biomarkers for the earliest stages of Alzheimer’s disease. Nevertheless, this study was cross-sectional, and examined only the prodromal stage of Alzheimer’s disease. Longitudinal studies are required to examine more carefully the temporal progression of network impairments that our model proposes, and to test whether the relationship between amyloid-β-pathology and intrinsic connectivity holds in later stages of the disease.

### Conclusion and outlook

In this study, we show evidence that amyloid-β-plaques accumulating in medial and lateral heteromodal fronto-parietal networks in prodromal Alzheimer’s disease have a robust impact on intrinsic functional connectivity at the local scale, and that their accumulation focuses on network cores and declines toward the periphery of networks. These results led us to an extension of the network degeneration hypothesis. It supposes that amyloid-β deposition and functional impairment spread by the same mechanism in many networks, but that the onset is graded such that it affects the DMN more strongly than others, and affects cores more strongly than (and possibly before) peripheries. Since our model predicts that the same mechanism governs amyloid-β-related neurodegeneration, irrespective of the affected network, it should also apply to other variants of Alzheimer’s disease, such as posterior cortical atrophy or logopenic primary progressive aphasia (Lehmann *et al.*, 2013*a*, *b*). In this framework, amyloid-β pathology would spread out by the same mechanism, but begin in different networks. Future studies examining this possibility could help in the development of mechanistic accounts of neurodegeneration.

## Supplementary Material

Supplementary Data

## Abbreviations

ATN: attention network
DMN: default mode network
PiB: Pittsburgh compound B
