# Supplementary material for: Within-patient correspondence of amyloid-β and intrinsic network connectivity in Alzheimer’s disease
Source: Brain. 2014 Apr 26;137(7):2052–64. doi: 10.1093/brain/awu103 (PMC4065018; doi:10.1093/brain/awu103)
Supplement: Supplementary Data [file supp_137_7_2052__index.html]

Within-patient correspondence of amyloid-β and intrinsic network connectivity in Alzheimer’s disease — Supplementary Data 

# Within-patient correspondence of amyloid-β and intrinsic network connectivity in Alzheimer’s disease

## Supplementary Data

files

**Files in this Data Supplement:**

- Supplementary Data - docx file
