# Supplementary material for: Within-patient correspondence of amyloid-β and intrinsic network connectivity in Alzheimer’s disease
Source: Brain. 2014 Apr 26;137(7):2052–64. doi: 10.1093/brain/awu103 (PMC4065018; doi:10.1093/brain/awu103)
Supplement: Supplementary Data [file supp_awu103_brain-2013-02137-File006.docx]

# *Supplemental Materials for*

**”Within-patient correspondence of amyloid-β and intrinsic network connectivity in Alzheimer’s disease“**

by Myers et al.

***Supplemental Methods***

Analysis of Gray Matter Differences (voxel-based morphometry)

Control Analysis Accounting for Differences in Group Size

***Supplemental Results (including Supplemental Figures):***

- Frontoparietal heteromodal intrinsic networks in healthy controls and patients (including ***Fig. S1*** Comparison of global and local network intrinsic connectivity)

- Gray matter differences in patients (including ***Fig. S2*** Voxel-based morphometry shows that group differences in gray matter density are restricted mainly to medial temporal regions)

- Control analyses of r_LOCAL_ accounting for orthogonalization procedure (including ***Fig. S3*** Supplemental and control analyses of r_LOCAL_)

- Control Analysis Accounting for Differences in Group Size

- Mean standardized uptake value (SUV) for PiB-PET (including ***Fig. S4*** Mean standardized uptake value for large cortical volume of interest)

- Illustration of voxel-wise correlation between PiB-uptake and connectivity (including ***Fig. S5*** Example scatterplot illustrating voxel-wise correlation between PiB-uptake and connectivity)

***Supplemental Tables***

- Tables S1-7: Intrinsic network peak activity and group differences

- Table S8: Gray Matter Differences between Groups

- Table S9: Results of Subsampling Analysis

**Supplemental Methods**

***Analysis of Gray Matter Differences (using voxel-based morphometry, VBM)***

We were interested in whether reductions in gray matter volume in our networks of interest in the patient group could have a potentially confounding effect on our results. As described recently (Sorg et al., 2013), we used the VBM8 toolbox (http://dbm.neuro.uni-jena.de/vbm.html) to analyze brain structure. T1-weighted images were corrected for bias-field inhomogeneity, registered using linear (12-parameter affine) and nonlinear transformations, and tissue-classified into gray matter (GM), white matter (WM), and cerebro-spinal fluid (CSF) within the same generative model (Ashburner and Friston, 2005). The resulting GM images were modulated to account for volume changes resulting from the normalization process. Here, we only considered non-linear volume changes so that further analyses did not have to account for differences in head size. Finally images were smoothed with a Gaussian kernel of 8mm (FWHM). For group comparisons, voxel-wise two-sample t-tests were performed. We applied a height threshold (voxel level) of 0.001, uncorrected. Volumes of global GM and WM and of intrinsic networks of interest were derived from the first segmentation process.

***Control Analysis Accounting for Differences in Group Size***

The control group in our study was noticeably smaller than our patient group. To test whether differences in group sizes could affect our results, we conducting a subsampling analysis that tests for differences when group sizes are matched. For a given analysis (e.g., an ANOVA testing PiB-uptake between groups and across networks), after correcting for gray matter density, age, and gender, we randomly selected a number of patients equal to the number of controls. We then calculated statistics (e.g., F statistics and the corresponding p-values). Since this analysis was based on a random selection of patients, we repeated the sampling and analysis step 1000 times, and evaluated the results by calculating median p-values across the 1000 repetitions, as well as the frequency of finding a significant effect.

**Supplemental Results**

***Fronto-parietal heteromodal intrinsic networks in healthy controls and patients***

To investigate group differences in intrinsic functional connectivity for each network at global level, we extracted the median connectivity z-value across all network voxels. We used ANOVAs and post-hoc t-tests to compare these average z-values between groups (see Fig. S1a). While the modestly significant main effect of group indicated an overall reduction in connectivity in the patient group (F_1,33_ = 4.29, p = 0.046), no network-specific comparisons survived Bonferroni correction for 7 tests (all t_33_ < 2.29, all corrected p > 0.20). Without correction, only the right attention network (rATN) showed a decrease in connectivity (t_33_ = 2.29, p = 0.029).

Furthermore, we used conventional voxel-wise two-sample t-tests to test for reductions in intrinsic connectivity in the patient group, compared to controls. We found significant reductions only in two regions: connectivity z-values in the posterior DMN was reduced in the right precuneus, and for the right attention network in the right inferior parietal lobule. We found no other significant group differences (see Supplementary Tables S1-7 and Fig. S1b below).


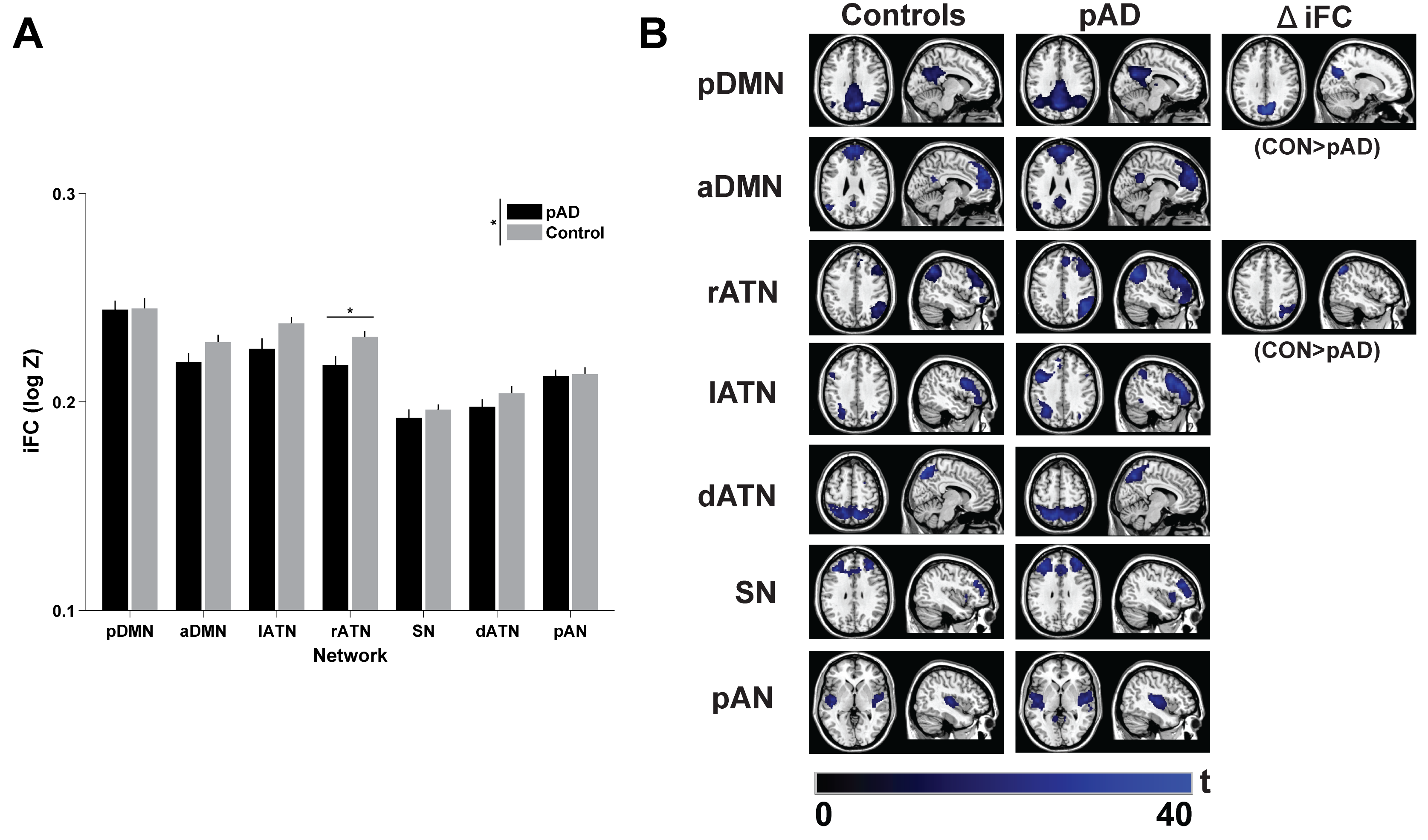


**Figure S1.** Comparison of global and local network connectivity z-values. **(A)** Global intrinsic connectivity (i.e. the median connectivity z-value of all voxels in the network) was not substantially decreased in patients, compared to controls. **(B)** Results of resting-state functional MRI of patients and controls. Intrinsic connectivity networks characterized by spatial patterns of functional connectivity of resting-state brain activity for patients and controls (SPMs of 1-sample t-tests for each network; p<0.05 FWE corrected at cluster level). Right column: SPMs showing locally decreased connectivity z-values in patients (two-sided two-sample t-tests; p<0.05 FWE cluster level, for illustration purposes, p<0.05, k=10). All SPMs are projected onto a single subject anatomical T1-weighted image. iFC: intrinsic functional connectivity, DMN: default mode network, p: posterior, a: anterior, ATN: attention network, r: right, l: left, d: dorsal, SN: salience network, pAN: primary auditory network. Colorscale indicates t statistics of one-sample (or two-sample, in the right column) t-tests.

***Gray matter differences in patients***


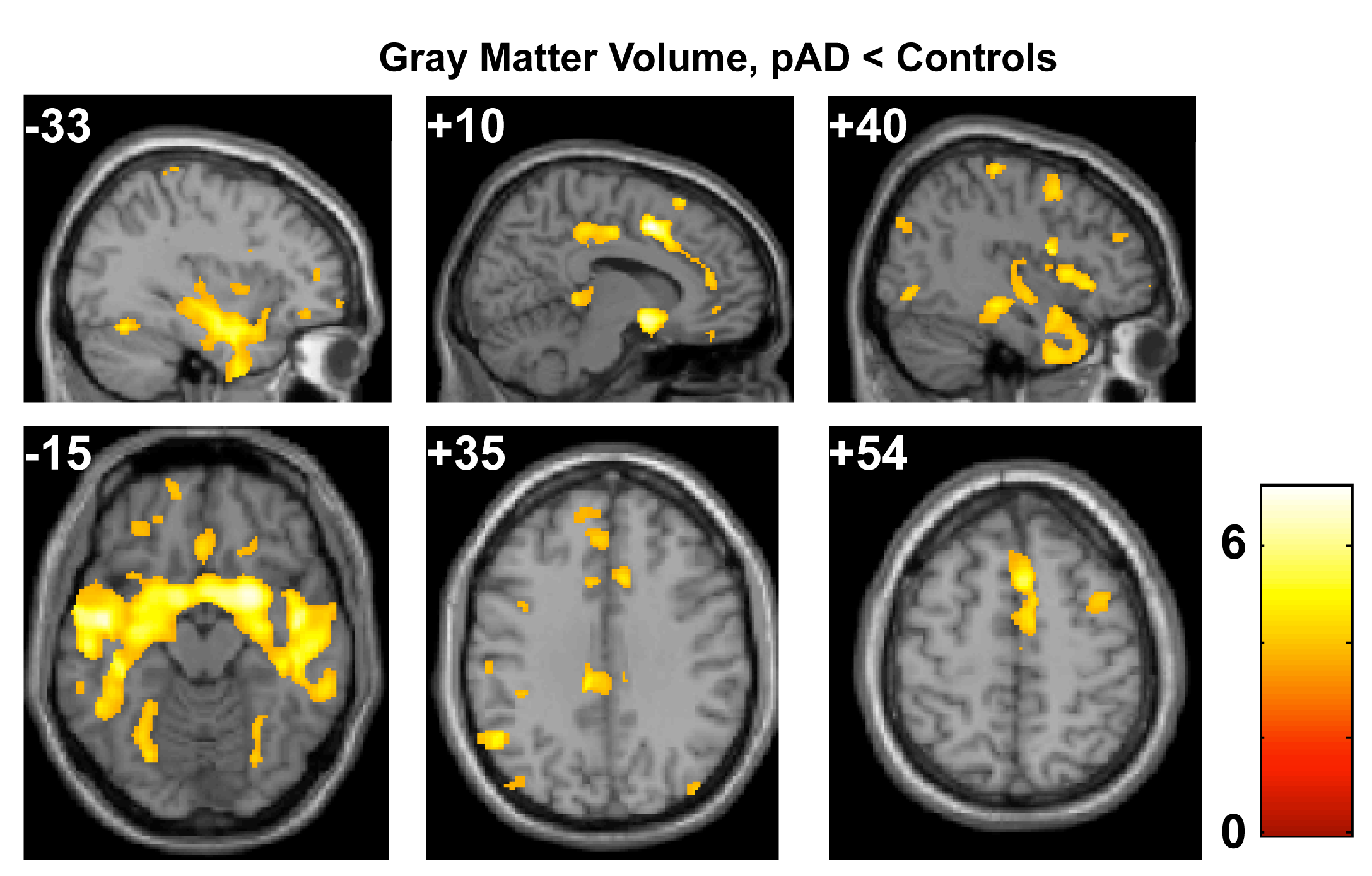


**Figure S2.** Voxel-based morphometry shows that group differences in gray matter density are restricted mainly to medial temporal regions. Plotted is a map of the t-test contrasting patient and control groups, thresholded at p < 0.001, uncorrected for multiple comparisons. The majority of group differences is restricted to the medial and lateral anterior temporal lobes, extending into the amygdala, ventral striatum, and anterior cingulate. Notably, there are only sparse GM differences in the lateral parietal and prefrontal cortex, as well as the posterior default mode regions overlapping with DMN and attention networks (also indicated in supplemental table S8).

***Control Analyses of r_LOCAL_ accounting for orthogonalization procedure***

In addition to the searchlight analyses described in the main text, we performed a control analysis to ensure that the orthogonalization step (which accounted for the effects of r_GLOBAL_) did not lead to any bias towards negative correlations. We calculated r_LOCAL_ for one subject and network for neighboring voxels (Fig. S3b, as in the analyses shown in Fig. 3) and for matched numbers of voxels sampled randomly from the entire network (Fig. S3b, bottom panel). In the shuffled dataset, the average r_LOCAL_ was close to 0, confirming that orthogonalization did not bias the analysis.

To further illustrate its necessity, we also calculated r_LOCAL_ for all subjects in one network (pDMN) with and without orthogonalization (Fig. S3c). Without orthogonalization, we found no strong positive or negative r_LOCAL_ anywhere in the network. Likewise, subtracting r_GLOBAL_ from (the uncorrected) r_LOCAL_ led only to a small negative correlation in the center of the pDMN. This demonstrates that the proper correction for r_GLOBAL_ was necessary to reveal the local effects.

In a last step, we tested whether there was a relationship between the absolute level of PiB-uptake and the impact it has on r_LOCAL_ (Fig. S3a). Here, for each network and subject, we used linear regression of PiB-uptake on r_LOCAL_ across all network voxels. As before, we submitted the resulting regression weights to two-way mixed-effects ANOVA. We found that in regions with high PiB-uptake, amyloid-β also tended to have a stronger effect on intrinsic connectivity (i.e. r_LOCAL_ was more negative, as measured by linear regression). This effect was significant for all networks except pAN and dATN (main effect group, F_1,33_ = 34.86, p < 10^-5^, network-wise t-tests between MCI and controls, all t_33_ < -3.27, corrected p < 0.018, dATN, t_33_ = -2.61, p = 0.094, pAN, t_33_ = -1.99, p = 0.393).


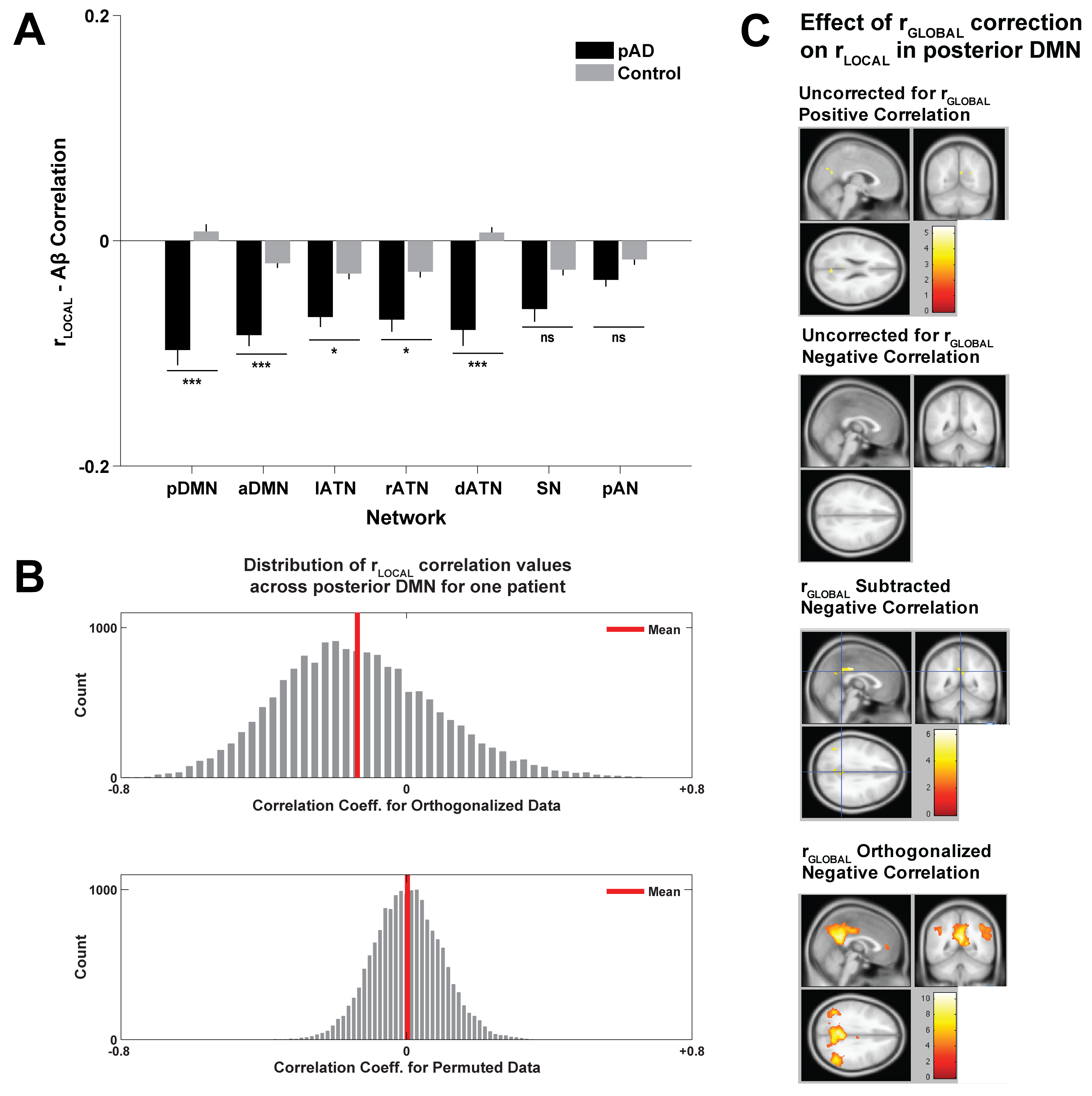


**Figure S3.** Supplemental and Control Analyses of r_LOCAL_. **(A)** Negative impact of amyloid-β on intrinsic connectivity is most pronounced in high-PiB regions. After calculating r_LOCAL_ (see Fig. 1&3) in every network voxel, we used linear regression to estimate the correlation between voxel-wise PiB-uptake and r_LOCAL_. The resulting regression coefficient quantifies how much the local negative impact of PiB on connectivity z-values depends on overall PiB levels in that neighborhood. These regression coefficients, estimated separately for every subject and network, were then submitted to mixed-effects ANOVAs and post-hoc t-tests. **(B)** Control Analysis of the Effect of Orthogonalization. To ensure that orthogonalizing intrinsic connectivity z-values and PiB-uptake across the entire network did not lead to a bias towards negative correlations, we calculated r_LOCAL_ for one subject for neighboring voxels (top panel, as in the analyses shown in Fig. 3) and for matched numbers of voxels sampled randomly from the entire network (bottom panel). In the shuffled dataset, the distribution of r_LOCAL_ was centered on 0, confirming that orthogonalization did not bias the analysis. **(C)** Effect of orthogonalization on r_LOCAL_ at the group level in one network. For the pDMN, we calculated the average local PiB-uptake/connectivity z-values correlation before and after accounting for r_GLOBAL_ through orthogonalization. Top panels show no significant (positive or negative) local correlations in the pDMN, across the pAD group. Subtracting r_GLOBAL_ from r_LOCAL_ (bottom left panel) led to a small negative correlation in the posterior cingulate. However, this negative correlation was significantly stronger after orthogonalizing across the network (bottom right panel). Maps show t-statistics from a one-sample t-test (thresholded at p<0.001, uncorrected) on the Fisher-transformed local correlation values (calculated at the single subject level). Color scale of the t-maps is indicated next to each panel, except for the top right, which showed no suprathreshold voxels.

***Control Analysis Accounting for Differences in Group Size***

The table below (S9) details the effects of reducing the patient group size (via subsampling) to match the size of the control group. Each column shows the statistical outcome of between-group comparisons across all networks (first column, main effect of group in a mixed-model ANOVA with factors group and network) or in a single network (two-sample t-test). For each outcome measure (PiB-uptake, r_GLOBAL_, and median r_LOCAL_, in different rows), we ran statistics on 1000 random subsamplings of the patient group (each time matching the size of the control group). On each random subsampling and statistical test (ANOVA or t-test), we calculated a p-value, generating 1000 p-values for each comparison. Shown are the median p-values (top rows) and the percentage of significant tests (p<0.05) out of the 1000 subsamplings. For t-tests, we show Bonferroni-corrected values (corrected for 7 networks), along with uncorrected values underneath. Most comparisons that are significant in the main text remain significant (and for the control network, pAN, r_GLOBAL_ and r_LOCAL_ are still not significant, as expected). The only exceptions are r_GLOBAL_ and r_LOCAL_ in two networks (lATN and SN), and r_LOCAL_ in one network (rATN). These, however, are highly significant (median p<0.01) at the uncorrected level.

***Mean standardized uptake value (SUV) for PiB-PET.***


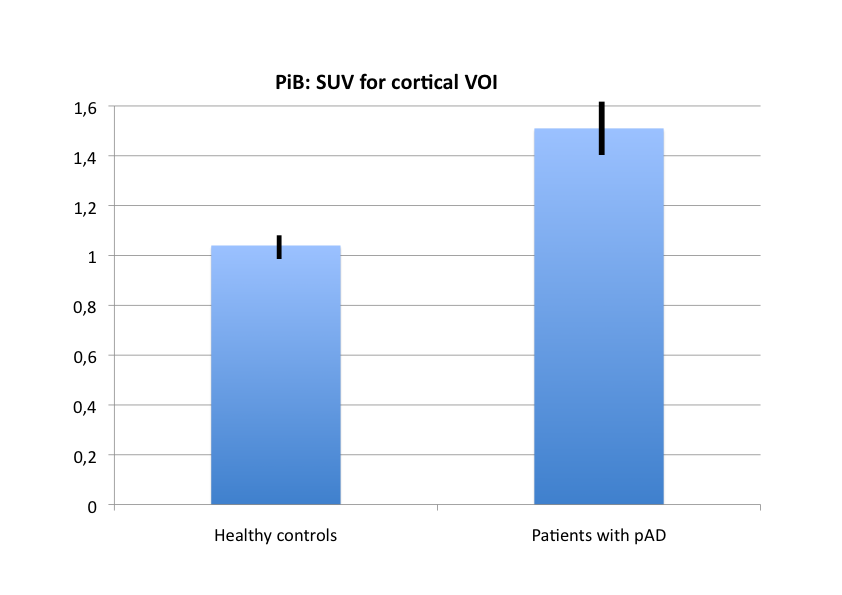


**Figure S4. Mean standardized uptake value (SUV) for large cortical volume of interest (VOI).** Mean PiB-uptake scores within pre-established large cortical volume of interest (VOI) including lateral prefrontal, parietal, and temporal areas and the retrosplenial cortex were used to define PiB-positivity and -negativity (Hedden et. al, 2009, Drzega et al., 2011). Cut-off for ‘high (i.e. PiB-positive)’ or ‘low (i.e. PiB-negative)’ neocortical standardized uptake value (SUV) ratios was 1.15 (Hedden et al, 2009, Drzezga et al., 2011). Patients were PiB-positive and healthy controls were PiB-negative.

***Illustration of voxel-wise correlation between PiB-uptake and connectivity.***

**
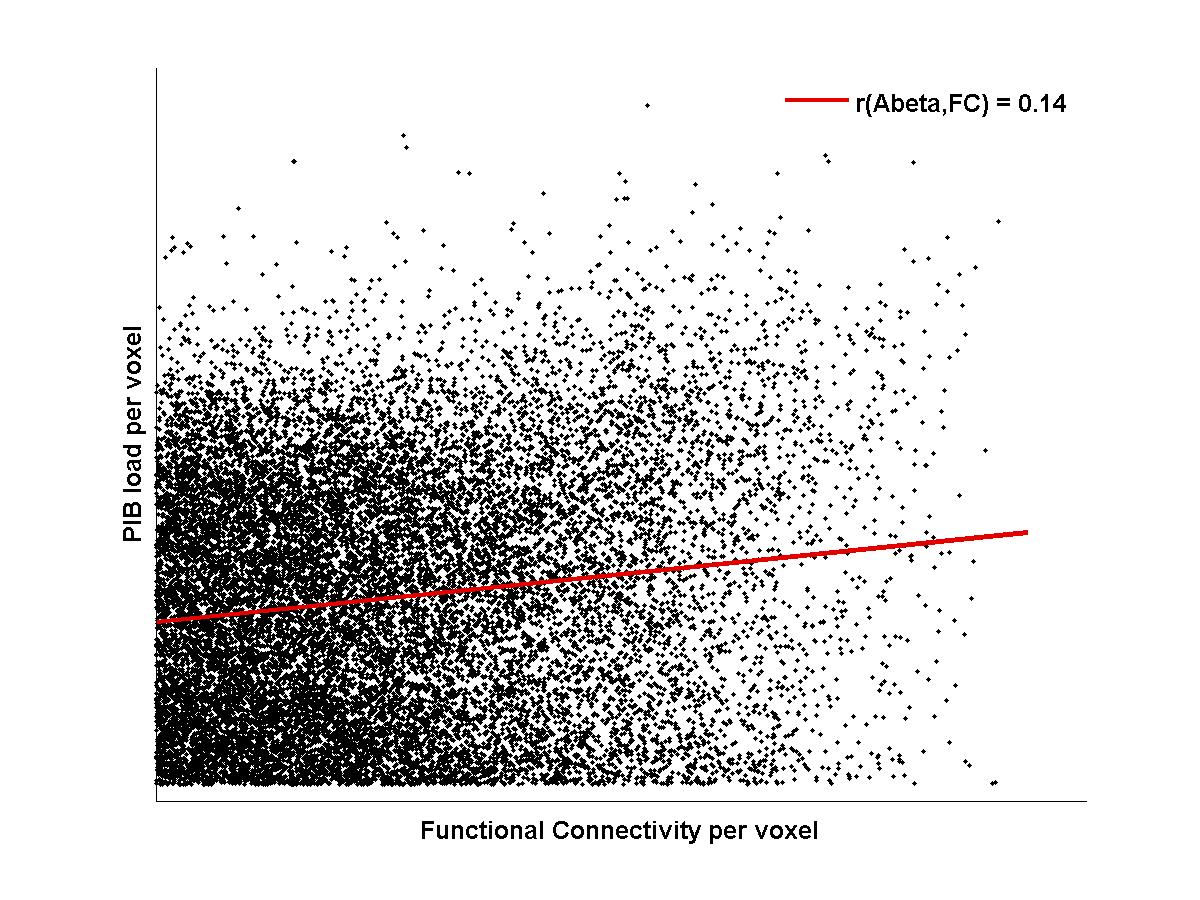
**

**Figure S5. Example scatterplot illustrating voxel-wise correlation between PiB-uptake and connectivity.** The plot shows all voxels taken from the posterior DMN of one patient (black dots). As can be seen, there is a modest positive correlation (Pearson r = 0.14, red line).

**Supplemental Tables**

### *Tables S1-7: Intrinsic network peak activity and group differences.*

***Table S1.***

| Posterior Default Mode Network | | | | | | | |
| --- | --- | --- | --- | --- | --- | --- | --- |
| Anatomical region | L/R | cluster | Z-score | | T-score | p-value | MNI (x;y;z) |
| Controls, one-sample-t-test | | | | | | | |
| Precuneus | L | 6195 | | >8 | 39.71 | <0.001 | -6;-66;28 |
| Precuneus | R |  | | >8 | 34.98 | <0.001 | 8;-62;32 |
| Posterior Cingulate Cortex | R |  | | 6.58 | 16.56 | <0.001 | 2;-32;36 |
| Posterior Cingulate Cortex | L |  | | 6.85 | 18.86 | <0.001 | -4;-44;32 |
| Inferior Parietal Lobule | R |  | | 7.61 | 27.78 | <0.001 | 40;-62;34 |
| Inferior Parietal Lobule | L | 521 | | 5.20 | 8.99 | 0.018 | -42;-60;28 |
| Anterior Medial Prefrontal Cortex | R | 4 | | 5.14 | 8.78 | 0.025 | 6;48;2 |
| Patients, one-sample-t-test | | | | | | | |
| Precuneus | L | 11051 | | >8 | 36.86 | <0.001 | -4;-68;32 |
| Precuneus | R |  | | >8 | 29.73 | <0.001 | 6;-66;34 |
| Posterior Cingulate Cortex | R |  | | >8 | 26.28 | <0.001 | 4;-44;24 |
| Posterior Cingulate Cortex | L |  | | >8 | 25.72 | <0.001 | -2;-56;30 |
| Inferior Parietal Lobule | L |  | | >8 | 19.82 | <0.001 | -40;-62;38 |
| Inferior Parietal Lobule | R |  | | 7.01 | 13.40 | <0.001 | 48;-60;28 |
| Anterior Medial Prefrontal Cortex | L | 10 | | 5.19 | 7.28 | 0.015 | -6;48;4 |
| Controls>Patients, two-sample-t-test | | | | | | | |
| Precuneus | R | 24 | | 4.14 | 4.69 | 0.022 | 8;-66;32 |

***Table S2.***

| Anterior Default Mode Network | | | | | | |
| --- | --- | --- | --- | --- | --- | --- |
| Anatomical region | L/R | cluster | Z-score | T-score | p-value | MNI (x;y;z) |
| Controls, one-sample-t-test | | | | | | |
| Superior Medial Gyrus | L | 5395 | 7.25 | 22.99 | <0.001 | -6;52;36 |
| Superior Medial Gyrus | R |  | 6.71 | 17.65 | <0.001 | 6;52;30 |
| Pregenual Anterior Cingulate Cortex | L |  | 7.07 | 21.02 | <0.001 | -4;50;12 |
| Pregenual Anterior Cingulate Cortex | R |  | 5.84 | 11.86 | <0.001 | 10;40;28 |
| Angular Gyrus | L | 309 | 6.75 | 17.97 | <0.001 | -54;-60;30 |
| Posterior Cingualte Cortex | L/R | 182 | 5.93 | 12.30 | <0.001 | +/- 2;-54;24 |
| Patients, one-sample-t-test | | | | | | |
| Superior Medial Gyrus | R | 7771 | >8 | 27.75 | <0.001 | 8;56;20 |
| Superior Medial Gyrus | L |  | >8 | 22.18 | <0.001 | -6;44;36 |
| Pregenual Anterior Cingulate Cortex | L/R |  | 7.42 | 15.48 | <0.001 | 0;42;12 |
| Posterior Cingualte Cortex | L | 861 | 6.94 | 13.09 | <0.001 | -2;-52;32 |
| Angular Gyrus | L | 395 | 6.17 | 10.06 | <0.001 | -52;-58;28 |

***Table S3.***

| Right Attention Network | | | | | | |
| --- | --- | --- | --- | --- | --- | --- |
| Anatomical region | L/R | cluster | Z-score | T-score | p-value | MNI (x;y;z) |
| Controls, one-sample-t-test | | | | | | |
| Inferior Parietal Lobule | R | 2234 | >8 | 40.74 | <0.001 | 44;-58;46 |
| Middle Frontal Gyrus | R | 2003 | 7.50 | 26.18 | <0.001 | 44;20;48 |
| Superior Frontal Gyrus | R | 930 | 7.04 | 20.71 | <0.001 | 22;60;6 |
| Medial Frontal Gyrus | R | 345 | 5.70 | 11.15 | 0.001 | 10;44;40 |
| Cerebellum | L | 52 | 5.47 | 10.09 | 0.004 | -10;-80;-30 |
| Patients, one-sample-t-test | | | | | | |
| Middle Frontal Gyrus | R | 7633 | 7.63 | 16.74 | <0.001 | 36;16;48 |
| Superior Frontal Gyrus | R |  | 6.48 | 11.17 | <0.001 | 16;34;60 |
| Medial Frontal Gyrus | R |  | 6.93 | 13.02 | <0.001 | 10;34;38 |
| Inferior Parietal Lobule | R | 2899 | >8 | 21.04 | <0.001 | 52;-56;50 |
| Cerebellum | L | 1068 | 7.05 | 13.57 | <0.001 | -34;-72;-44 |
| Controls>Patients, two-sample-t-test | | | | | | |
| Inferior Parietal Lobule | R | 19 | 4.15 | 4.70 | 0.044 | 50;-58;50 |

***Table S4.***

| Left Attention Network | | | | | | |
| --- | --- | --- | --- | --- | --- | --- |
| Anatomical region | L/R | cluster | Z-score | T-score | p-value | MNI (x;y;z) |
| Controls, one-sample-t-test | | | | | | |
| Inferior Parietal Lobule (Angular Gyrus) | L | 1715 | 7.25 | 22.87 | <0.001 | -52;-56;42 |
| Middle Frontal Gyrus | L | 575 | 7.14 | 21.74 | <0.001 | -38;28;46 |
| Superior Frontal Gyrus | L |  | 5.95 | 12.43 | <0.001 | -24;16;66 |
| Inferior Frontal Gyrus | L | 236 | 5.81 | 11.66 | <0.001 | -48;36;-16 |
| Inferior Parietal Lobule (Angular Gyrus) | R | 44 | 5.43 | 9.90 | <0.001 | 60;-44;38 |
| Patients, one-sample-t-test | | | | | | |
| Inferior Parietal Lobule (Angular Gyrus) | L | 2864 | >8 | 22.54 | <0.001 | -46;-62;52 |
| Superior and Middle Frontal Gyrus | L | 1755 | 7.45 | 15.68 | <0.001 | -42;20;48 |
| Inferior Frontal Gyrus | L | 579 | 6.83 | 12.58 | <0.001 | -40;46;-16 |
| Inferior Parietal Lobule (Angular Gyrus) | R | 903 | >8 | 19.62 | <0.001 | 56;-58;40 |
| Cerebellum | R | 960 | 6.64 | 11.80 | <0.001 | 38;-64;-40 |

***Table S5.***

| Dorsal Attention Network | | | | | | |
| --- | --- | --- | --- | --- | --- | --- |
| Anatomical region | L/R | cluster | Z-score | T-score | p-value | MNI (x;y;z) |
| Controls, one-sample-t-test | | | | | | |
| Superior and Inferior Parietal Lobule | L | 5849 | 7.64 | 28.15 | <0.001 | -30;-56;48 |
| Precuneus | L |  | 7.28 | 23.34 | <0.001 | -12;-72;46 |
| Precuneus | R |  | 7.45 | 25.45 | <0.001 | 12;-72;50 |
| Superior and Inferior Parietal Lobule | R |  | 7.24 | 22.93 | <0.001 | 22;-72;50 |
| Patients, one-sample-t-test | | | | | | |
| Superior and Inferior Parietal Lobule | R | 9743 | >8 | 27.08 | <0.001 | 24;-70;52 |
| Precuneus | R |  | >8 | 22.97 | <0.001 | 8;-64;50 |
| Superior and Inferior Parietal Lobule | L |  | >8 | 22.92 | <0.001 | -26;-64;54 |
| Precuneus | L |  | >8 | 19.88 | <0.001 | -4;-52;62 |

***Table S6.***

| Salience Network | | | | | | |
| --- | --- | --- | --- | --- | --- | --- |
| Anatomical region | L/R | cluster | Z-score | T-score | p-value | MNI (x;y;z) |
| Controls, one-sample-t-test | | | | | | |
| Middle and Superior Frontal Gyrus | L | 2094 | 7.23 | 22.81 | <0.001 | -36;36;32 |
| Anterior Cingulate Cortex | R |  | 6.13 | 13.48 | <0.001 | 8;32;20 |
| Middle and Superior Frontal Gyrus | R | 1045 | 6.50 | 16.00 | <0.001 | 32;40;28 |
| Insula Lobe | L | 239 | 6.73 | 17.77 | <0.001 | -32;16;10 |
| Superior Orbital Gyrus | R | 88 | 5.69 | 11.07 | 0.001 | 24;50;0 |
| Insula Lobe | R | 53 | 5.82 | 11.72 | 0.001 | 32;18;-8 |
| Patients, one-sample-t-test | | | | | | |
| Middle and Superior Frontal Gyrus | R | 6851 | 7.84 | 18.24 | <0.001 | 30;48;22 |
| Middle and Superior Frontal Gyrus | L |  | 7.77 | 17.69 | <0.001 | -26;48;14 |
| Anterior Cingulate Cortex | R |  | 6.47 | 11.14 | <0.001 | 6;32;32 |
| Insula Lobe | L |  | 6.84 | 12.63 | <0.001 | -32;12;6 |
| Insula Lobe | R | 329 | 5.76 | 8.77 | 0.001 | 36;24;-2 |

***Table S7.***

| Primary Auditory Network | | | | | | |
| --- | --- | --- | --- | --- | --- | --- |
| Anatomical region | L/R | cluster | Z-score | T-score | p-value | MNI (x;y;z) |
| Controls, one-sample-t-test | | | | | | |
| Insula/Superior Temporal Gyrus | R | 1894 | 7.38 | 24.52 | <0.001 | -42;-20;12 |
| Insula/Superior Temporal Gyrus | L | 1462 | 7.11 | 21.41 | <0.001 | 44;-20;8 |
| Anterior Cingulate Cortex | L/R | 294 | 5.93 | 12.31 | <0.001 | 0;20;16 |
| Patients, one-sample-t-test | | | | | | |
| Insula/Superior Temporal Gyrus | L | 3791 | >8 | 19.87 | <0.001 | -54;-30;8 |
| Insula/Superior Temporal Gyrus | R | 3067 | >8 | 20.50 | <0.001 | 60;-16;12 |
| Anterior Cingulate Cortex | L/R | 1370 | 7.09 | 14.62 | <0.001 | 0;24;30 |

One-sample-t-tests are thresholded at p<0.05 FWE corrected. Clusters of the two-sample-t-test are FWE corrected.

### *Table S8: Gray Matter Differences between Groups.*

| Anatomical region | L/R | cluster | Z-score | T-score | p-value | x (MNI) | y | z |
| --- | --- | --- | --- | --- | --- | --- | --- | --- |
| Controls > pAD, two-sample-t-test | | | | | | | | |
| cingulate gyrus | R | 13458 | 5.71 | 7.25 | <0.001 | 10 | 8 | 46 |
| superior / medial temporal gyrus | R | 56041 | 5.55 | 6.95 | <0.001 | 56 | -24 | -5 |
| postcentral gyrus | R | 878 | 4.52 | 5.24 | <0.001 | 45 | -25 | 63 |
| superior frontal gyrus | R | 799 | 4.22 | 4.81 | <0.001 | 30 | 58 | 1 |
| superior parietal lobule | L | 258 | 4.21 | 4.79 | <0.001 | -15 | -63 | 69 |
| postcentral gyrus | L | 434 | 4.19 | 4.77 | <0.001 | -44 | -18 | 61 |
| middle frontal gyrus | R | 362 | 4.16 | 4.72 | <0.001 | 36 | 42 | 22 |
| superior frontal gyrus | R | 472 | 4 | 4.5 | <0.001 | 18 | 45 | 45 |
| angular gyrus | L | 385 | 4 | 4.49 | <0.001 | -46 | -79 | 30 |
| superior orbitofrontal gyrus | R | 907 | 3.94 | 4.41 | <0.001 | 18 | 34 | -26 |
| superior frontal gyrus | R | 186 | 3.85 | 4.29 | <0.001 | 20 | 57 | 27 |
| fusiform gyrus | R | 314 | 3.85 | 4.29 | <0.001 | 27 | -73 | -17 |
| middle temporal gyrus | R | 513 | 3.69 | 4.08 | <0.001 | 44 | -82 | 33 |
| superior frontal gyrus | R | 147 | 3.66 | 4.04 | <0.001 | 15 | 62 | -9 |
| inferior frontal gyrus | R | 278 | 3.61 | 3.98 | <0.001 | 57 | 14 | 9 |
| superior temporal gyrus | R | 148 | 3.55 | 3.89 | <0.001 | 56 | -27 | 16 |
| middle occipital gyrus | L | 185 | 3.54 | 3.88 | <0.001 | -30 | -81 | 18 |
| precentral gyrus | R | 81 | 3.51 | 3.85 | <0.001 | 48 | -16 | 39 |
| precuneus | R | 67 | 3.48 | 3.8 | <0.001 | 10 | -54 | 22 |
| middle occipital gyrus | R | 226 | 3.48 | 3.8 | <0.001 | 20 | -96 | 6 |
| cuneus | L | 56 | 3.46 | 3.79 | <0.001 | -14 | -100 | 19 |
| cerebellum | R | 260 | 3.41 | 3.72 | <0.001 | 20 | -79 | -47 |
| precuneus | L | 71 | 3.37 | 3.66 | <0.001 | -6 | -55 | 27 |

***Table S9. Results of Subsampling Analysis***

|  | main effect | pDMN | aDMN | lATN | rATN | SN | dATN | pAN |
| --- | --- | --- | --- | --- | --- | --- | --- | --- |
| **PiB** | | | | | | | | |
| median p (corr.) | <0.001 | 0.003 | 0.002 | 0.003 | 0.004 | 0.003 | 0.002 | 0.003 |
| % < 0.05 (corr.) | 100% | 99.8% | 100% | 99.7% | 98.4% | 99.3% | 100% | 99.6% |
| r_GLOBAL_ | | | | | | | | |
| median p (corr.) | <0.001 | <0.001 | 0.002 | 0.012 | 0.006 | 0.024 | <0.001 | 0.594 |
| % < 0.05 (corr.) | 100% | 100% | 100% | 85.7% | 94.4% | 65.9% | 100% | 10.8% |
| median p (uncorr.) |  | <0.001 | <0.001 | <0.001 | <0.001 | 0.004 | <0.001 | 0.080 |
| % < 0.05 (uncorr.) |  | 100% | 100% | 100% | 100% | 98.9% | 100% | 36.7% |
| r_LOCAL_ | | | | | | | | |
| median p (corr.) | <0.001 | <0.001 | 0.006 | 0.018 | 0.027 | 0.065 | <0.001 | 0.999 |
| % < 0.05 (corr.) | 100% | 100% | 93.3% | 73.5% | 65.3% | 42.5% | 100% | 0% |
| median p (uncorr.) |  | <0.001 | 0.001 | 0.003 | 0.004 | 0.009 | <0.001 | 0.550 |
| % < 0.05 (uncorr.) |  | 100% | 100% | 99.7% | 98.5% | 91.2% | 100% | 0% |

**Supplemental References**

Ashburner, J., and Friston, K.J. (2005). Unified segmentation. *NeuroImage* *26*, 839–851.

Drzezga, A., Becker, J.A., Van Dijk, K.R.A., Sreenivasan, A., Talukdar, T., Sullivan, C., Schultz, A.P., Sepulcre, J., Putcha, D., and Greve, D. (2011). Neuronal dysfunction and disconnection of cortical hubs in non-demented subjects with elevated amyloid burden. *Brain* *134*, 1635–1646.

Hedden, T., Van Dijk, K.R.A., Becker, J.A., Mehta, A., Sperling, R.A., Johnson, K.A., et al. (2009). Disruption of functional connectivity in clinically normal older adults harboring amyloid burden. *Journal of Neuroscience 29*, 12686–12694.

Sorg, C., Manoliu, A., Neufang, S., Myers, N., Peters, H., Schwerthöffer, D., et al. (2013). Increased intrinsic brain activity in the striatum reflects symptom dimensions in schizophrenia. *Schizophrenia Bulletin* *39*, 387–395.
